# Draculab: A Python Simulator for Firing Rate Neural Networks With Delayed Adaptive Connections

**DOI:** 10.3389/fninf.2019.00018

**Published:** 2019-04-02

**Authors:** Sergio Verduzco-Flores, Erik De Schutter

**Affiliations:** Computational Neuroscience Unit, Okinawa Institute of Science and Technology, Okinawa, Japan

**Keywords:** neural simulator, firing rate activity, Python, transmission delay, adaptive synapses

## Abstract

Draculab is a neural simulator with a particular use scenario: firing rate units with delayed connections, using custom-made unit and synapse models, possibly controlling simulated physical systems. Draculab also has a particular design philosophy. It aims to blur the line between users and developers. Three factors help to achieve this: a simple design using Python's data structures, extensive use of standard libraries, and profusely commented source code. This paper is an introduction to Draculab's architecture and philosophy. After presenting some example networks it explains basic algorithms and data structures that constitute the essence of this approach. The relation with other simulators is discussed, as well as the reasons why connection delays and interaction with simulated physical systems are emphasized.

## 1. Introduction

When faced with a new project, modelers in computational neuroscience must decide whether to program their simulations from scratch, or to use one of the existing neural simulators. Draculab was born from a project whose requirements were not met by any other simulator. Those requirements were:

- Firing rate units that operate as continuous-time dynamical systems, connected with transmission delays.
- Experimental types of units and synapses, with frequent modifications happening.
- Simulations where neural controllers interact with a physical system, implementing closed-loop control.

Besides satisfying these requirements, Draculab aims to have a simple interface, but still provide total control over the simulation to experienced Python users. For users with basic command of Python, or in a hurry to simulate, Draculab is a neural simulator with an interface similar to PyNEST (Eppler et al., 2009). For more experienced users, it is also a simulator where almost every aspect of the models can be customized.

Following Brian's (Goodman and Brette, 2009) insight that the main limiting factor is not always computational efficiency, Draculab is written entirely in Python, with certain key functions coded in Cython (Behnel et al., 2011) (https://cython.org/) for speed. Development environments such as Spyder (https://www.spyder-ide.org/), or the Jupyter Notebook (https://jupyter.org/), are well suited for working with Draculab. The interface uses standard functions that create units, connect them, and run simulations. These functions are configured using parameter dictionaries as their arguments. Users can launch their first simulations within minutes (see section 2).

It is expected that people who want to write a simulation do not want to learn a new programming language, or the intricacies of a complex interface. On the other hand, some users want to write very non-standard simulations, using their own synaptic plasticity rules, or their own firing rate models, perhaps connecting them with other dynamical systems. The idea to resolve this is to take advantage of the users' knowledge of Python. People who already understand Python do not have to learn many new things, except for the basic architecture of the system, which is described in the sections below.

For experienced Python users, Draculab aims to give ample control over the simulation, so it feels like they wrote the simulator themselves (but without going through the hassle of writing and testing it). This comes from a straightforward implementation, profusely commented source code, and standard libraries such as Scipy (https://www.scipy.org/), Numpy (https://www.numpy.org), and Matplotlib (https://matplotlib.org/) for numerical routines and visualization.

Section 2 introduces some examples so the reader can become acquainted with Draculab's API, and some use scenarios. Section 3 briefly explains the problem of numerically solving delay differential equations, and the approach followed by Draculab. Section 4 gives some details on how this approach is implemented using Python's data structures. Section 5 explains how to modify this implementation to increase simulation speed. Section 6 is concerned with how noise can be included in simulations. Section 7 mentions several tools that can be used to simplify the construction and analysis of Draculab networks. Finally, section 8 gives an overview of alternatives to Draculab, and how they compare.

Readers who want to proceed further can work through the tutorials included with the Draculab distribution (https://gitlab.com/sergio.verduzco/draculab), or consult the docstring documentation. Readers who only want a quick description of Draculab can skip the more technical sections 3, 4, and 5.

## 2. Example Networks

This section presents two Draculab simulations.

### 2.1. A First Example

To begin with, let's create a network with 10 sigmoidal units and one input. It does nothing special, but it illustrates the basic interface of the simulator.

We segment the source code of this example code into 5 steps, and analyze each one. The full program (in Jupyter notebook format) can be found in the file tutorial/hello_world.ipynb of the distribution.

**Figure 1:**
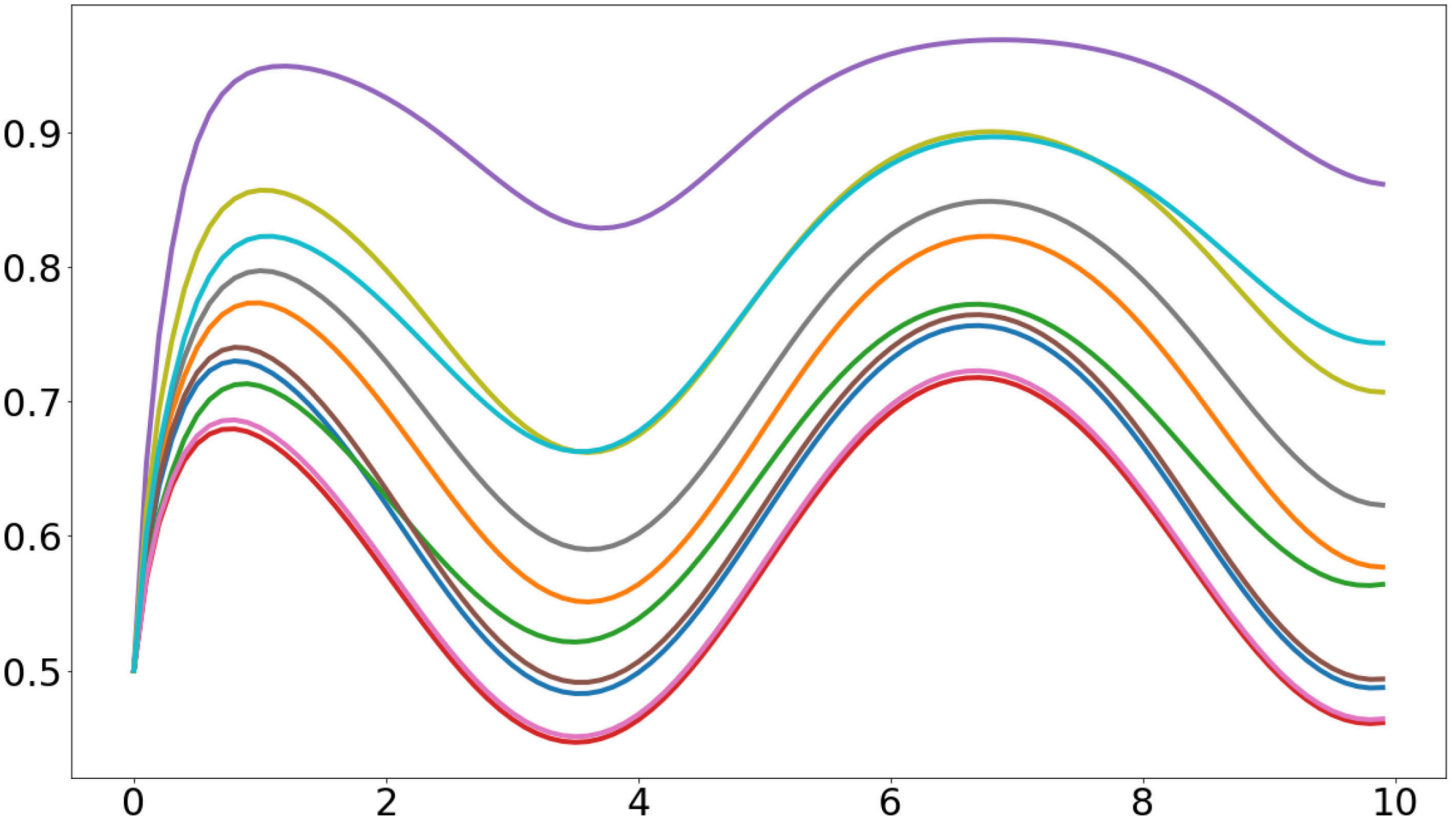
Output of the first example program. It can be seen that the heterogeneous initial values of the synaptic weights cause the units to have different responses.

1. Create a network object
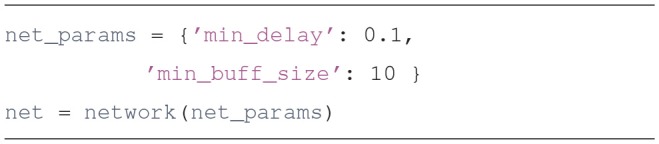
  The basic object used to run Draculab simulations is an instance of the class network. The constructor of this class requires a parameter dictionary with the smallest delay among all the connections (min_delay), and the number of values to be stored by each unit in the timespan of the smallest delay (min_buff_size). The significance of these parameters will be explained in section 3.
2. Populate the network with two types of units
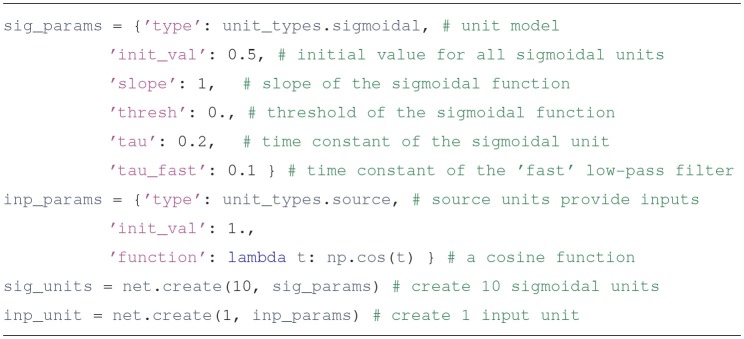
  First we create parameter dictionaries that configure the sigmoidal and the input units. All units have type and init_val attributes; other parameters to include depend on the type of the unit. The specifics can be consulted in the unit's documentation. For example, the Python command help(sigmoidal.__init__) will show the entries expected in the parameter dictionary for sigmoidal units.
  Usually units produce an output based on their inputs and their current state. source units are different, since their output comes from a Python function defined by the user, whose argument is the simulation time. This is a flexible way to specify inputs. Source units can also be used to report the value of any variable in the simulation (such as the synaptic weights) as it evolves through time.
  Upon creation, each unit is assigned an integer that uniquely identifies it. The create method returns a list with the identifiers of the created units.
3. Connect the units
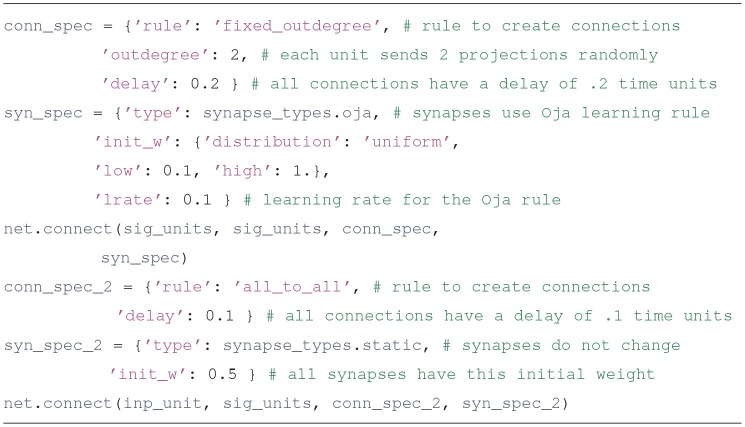
  Users familiar with PyNEST may find many similarities in how the connect method works. PyNEST was used as a template on how to specify connections, and Draculab has a topology module that resembles the one in PyNEST, although they are not identical (see section 7). In the example code, we create parameter dictionaries to configure the connections, and then call the connect method to create them. Running the Python instruction help(network.connect) can yield more details.
  In the syn_spec dictionary it can be observed that the synapses used for connections between sigmoidal units are of the oja type. The *Oja* learning rule (Oja, 1982) is a Hebbian-type model, usually associated with principal component extraction. In Draculab a continuous-time version of this model is implemented in the oja_synapse object. The network.connect function creates a synapse object for each connection. The name of all implemented synapse models can be obtained by typing the command synapse_types.list_names().
4. Run the simulation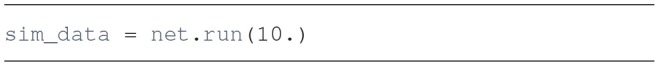  The run method receives a time to simulate, and returns a tuple containing simulation data. Calling run repeatedly restarts the simulation from its last time point. It should be noted that in Draculab no particular units of time, distance, or firing rate are enforced.
5. Plot the results
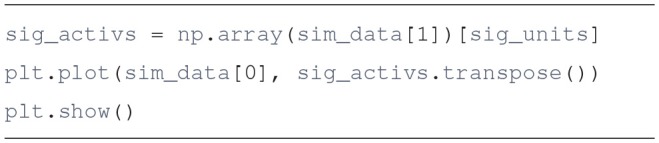
  In here we use the plot function from Matplotlib to visualize the activities of the 10 sigmoidal units. The resulting plot is similar to the one presented in Figure 1.

### 2.2. A Closed-Loop Simulation

**Figure 2:**
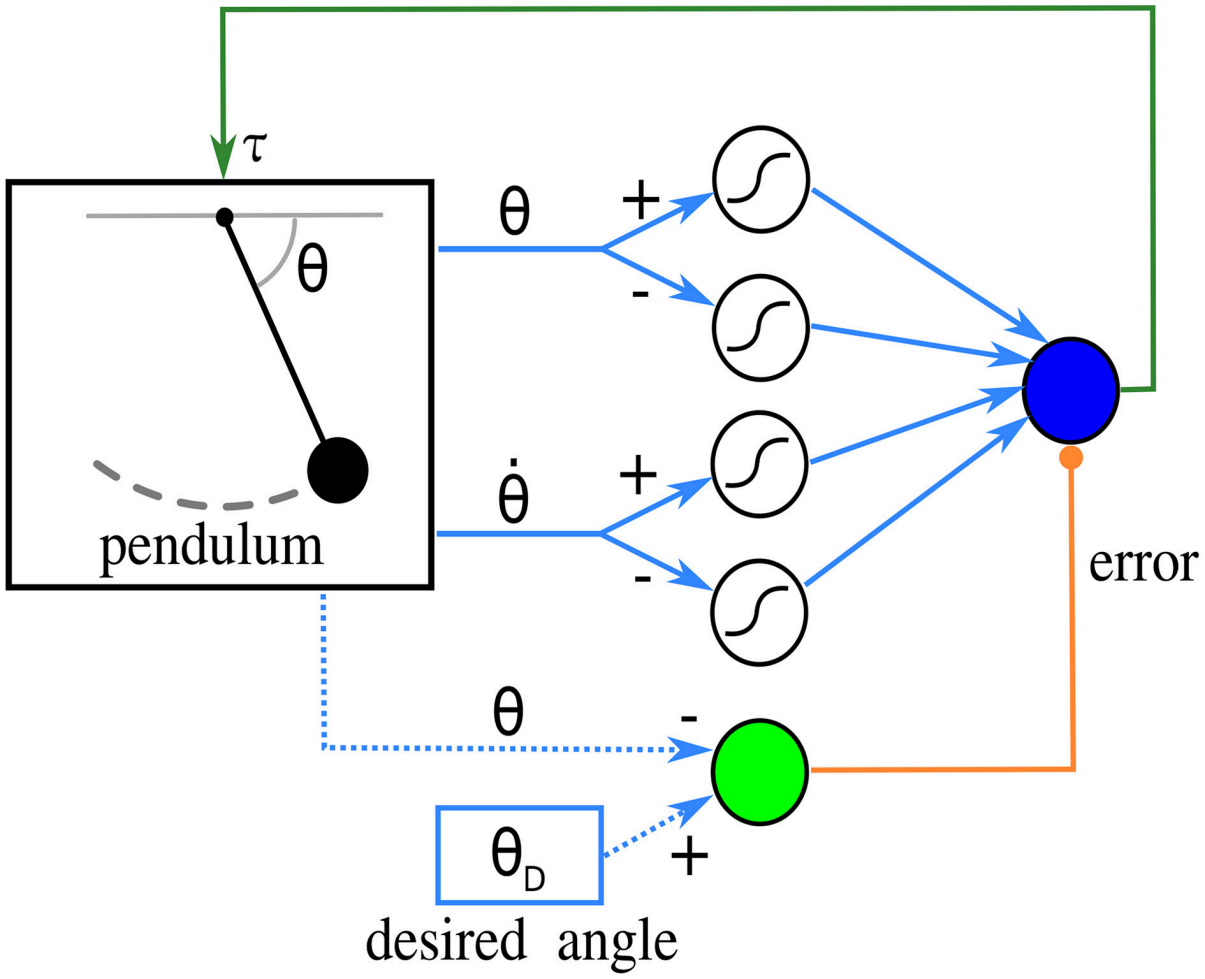
Diagram of the network in the closed-loop simulation. The pendulum sends angle and angular velocity signals to four sigmoidal afferent units, which in turn send inputs to a control unit (blue circle). The control unit produces a torque on the pendulum based on this input. The source unit, in green, calculates an error signal from the desired and the current angle. The derivative of this error is used by the learning rule. Blue dotted lines indicate that the source unit does not receive the angles from connections; instead, its function reads them directly.

Next is one of the simplest networks that perform feedback control of a physical system, with synaptic plasticity enhancing performance. Borrowing terminology from control engineering, the system to be controlled is called a *plant*. The plant is a pendulum, and a single linear *control* unit acts as a proportional feedback controller that puts the pendulum at a desired angle. The control unit receives an error signal from a source unit, and the state of the pendulum from four *afferent* sigmoidal units. Learning takes place at the synapses connecting afferent units to the control unit, using the *input correlation* rule (Porr and Wörgötter, 2006). The connectivity of this network can be observed in Figure 2.

The input correlation rule is defined by the following equation:

$$\begin{array}{l} \frac{d\omega_{j}}{dt}=\mu u_{j}\frac{du_{0}}{dt}, \end{array}$$

where ω_*j*_ is the weight of the input from the *j*-th unit, which has activity *u*_*j*_. Unit 0 provides an error signal, and by using its derivative the input correlation rule seeks to exploit correlations between input signals and error increase. This is implemented as the inp_corr synapse type.

Further details about this network and the full source code can be found in Draculab's tutorial, in the tutorial/tutorial4.ipynb file. In here we just briefly describe what was done, and the ensuing results.

**Figure 3:**
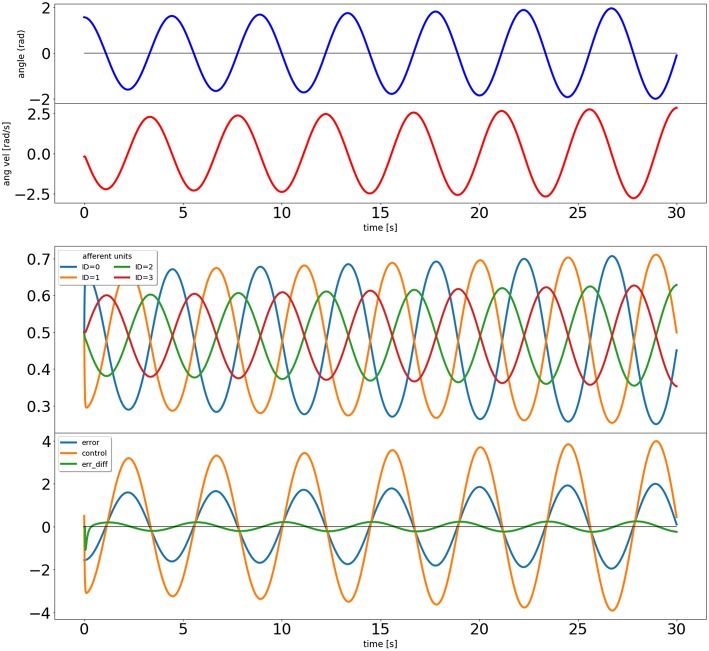
Output of the closed loop simulation using static synaptic weights. The top two plots show the angle and angular velocity of the pendulum. The next plot shows the activity of the four afferent units, and the bottom plot shows the error signal, the activity of the control unit, and a signal proportional to the error's derivative (used in the learning rule).

**Figure 4:**
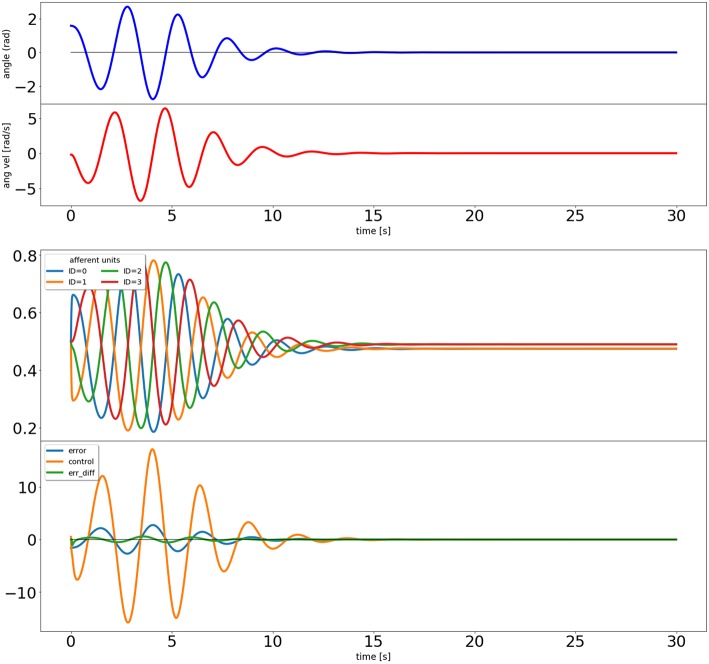
Output of the closed loop simulation using the input correlation rule to adjust the synaptic weights of the afferent inputs to the control unit.

When using the Draculab module in Python, a useful approach is to create a class that contains all the parameter dictionaries. This class can also be provided with methods to run the simulation, and to visualize the results. In this case we created a class called ic_control, whose constructor contains all the parameter dictionaries. These parameters can be modified by the user before calling the ic_control.initialize method, which creates the network, the pendulum, all units, and all connections. The pendulum is a special type of object that is derived from the plant class, which is used to model any dynamical system governed by ordinary differential equations. After initialize we can call the ic_control.simulate method, which receives a simulation time, runs the network for that time span, and reports the real time required to finish. Finally we can call ic_control.plot_results, which produces figures illustrating the simulation results (e.g., Figures 3, 4).

Creating the network for this example uses the same procedures as in the previous example. The one unfamiliar thing may be that the pendulum is connected to the units not with network.connect, but with the methods network.set_plant_inputs and network.set_plant_outputs.

Both units and plants may receive qualitatively different types of inputs. For example, units may have inputs that have a modulatory, rather than excitatory effect. Moreover, plants may have separate inputs that act on different state variables, such as torques on different joints of a double pendulum. To handle this Draculab uses *input ports*, which is a concept inspired by the NEST simulator (Diesmann and Gewaltig, 2001). The syn_spec parameter dictionary of the network.connect method can specify an input port, which gets stored in the synapse object. How different ports are interpreted depends on the particular unit and plant models, opening many possibilities, such as inputs that target distinct regions of the dendritic tree (e.g., London and Häusser, 2005).

We use the ic_control object to compare the performance of the network with and without the input correlation rule. First we set the learning rate to zero so the control unit becomes a proportional controller, only driven by the error:


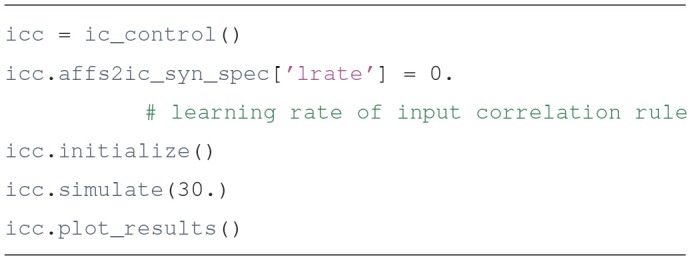


The results of this simulation can be seen in Figure 3. It is clear that the system is not stable, as would be expected from a proportional controller in an underdamped system. Next we simulate with a non-zero learning rate for the input correlation rule, obtained by replacing the second line of the code above by icc.affs2ic_syn_spec['lrate'] = 40.

The results can be seen in Figure 4. The input correlation rule is surprisingly effective, despite not using input filter banks (Porr and Wörgötter, 2006).

## 3. The Draculab Approach to Neural Simulation

A Draculab network is a collection of interconnected units, optionally interacting with a plant.

With the exception of *source* units, all units are continuous-time dynamical systems arising from an ordinary differential equation (ODE). Although a unit's ODE may be multidimensional, its output is always one-dimensional. Connections between units have a temporal delay and a synapse type. Temporal delays are multiples of a minimum delay. Synapses can too be dynamical systems, modifying their weights in response to presynaptic and postsynaptic events.

Plants are continuous dynamical systems that can be modeled with ODEs. Like units, they can receive input connections from multiple units, and unlike units they can produce several output values. Plants can be used to model a physical system that is being controlled by the units in the network.

In mathematical terms, a Draculab network is a first-order system of Delay Differential Equations (DDEs) of the form:

$$\begin{array}{l} \dot{u}\left(t\right)=f\left(t,u\left(t\right),u\left(t-\tau_{1}\right),u\left(t-\tau_{2}\right),\ldots ,u\left(t-\tau_{k}\right)\right), \end{array}$$

where *u*(*t*) ∈ ℝ^*n*^ is a state vector that includes the state variables for all units, plants, and synapses. The constants τ_*i*_ correspond to the connection delays, which explains the dependence on the *u*(*t* − τ_*i*_) functions.

An initial value problem (IVP) for this system involves finding a solution *u*(*t*) for *t* > *t*_0_ ∈ ℝ given an initial state ϕ(*t*) for *t* ∈ [*t*_0_ − τ, *t*_0_], with τ being the largest τ_*i*_ value. Notice that an IVP for a finite-dimensional ODE requires an initial state that is a finite-dimensional vector, whereas the DDE IVP requires a function ϕ:ℝ → ℝ^*n*^ defined in the interval [*t*_0_ − τ, *t*_0_] as its initial condition. In other words, the state of the DDE at any time *t*_0_ is the full trajectory in state space for the time interval [*t*_0_ − τ, *t*_0_], which is contained in the ϕ function.

The type of DDE that Draculab must solve, shown in the equation above, is relatively well-understood (Bellen and Zennaro, 2013). Moreover, there are well-established numerical methods for solving IVPs for this type of equation (Shampine and Thompson, 2009; Bellen and Zennaro, 2013). The simplest and most common numerical approach may be to adapt linear multistep methods for ODEs. Linear multistep methods for an ODE system $\dot{u}=f\left(t,u\right)$ use an iterative procedure where given the state *u*(*t*_*n*_) we find the state at the next time point *t*_*n* + 1_ with a formula *u*(*t*_*n* + 1_) = *u*(*t*_*n*_) + *L*, where *L* is a linear combination of values of *f* evaluated at intermediate time points in [*t*_*n*_, *t*_*n* + 1_]. This includes the very common Euler and Runge-Kutta methods.

As an illustrative example, we can adapt the forward Euler method to find an approximate solution of the system $\dot{u}\left(t\right)=1+u\left(t-1\right)$, with initial conditions *u*(*t*) = 0 for *t* ∈ [−1, 0]. Using a timestep Δ*t* = 0.1 we can begin with *u*(0) = 0, and find *u*(0.1) = *u*(0) + Δ*t*(1 + *u*(−1)) = 0.1. Repeating this procedure iteratively we find *u*(*nΔt*) = *nΔt* for *n* = 0, 1, …, 10. At this point our first calculated value enters the right-hand side of the equation, so at the next iteration we have *u*(1.1) = *u*(1) + Δ*t*(1 + *u*(0.1)) = 1.11. It is clear that we can continue this process at time *t*_*n*_ as long as we maintain in memory the values of *u*(*t*) for *t* = *t*_*n*_ − Δ*t, t*_*n*_ − 2Δ*t*, …, *t*_*n*_ − 10Δ*t*.

Draculab uses the approach of adapting linear multistep ODE numerical methods, which for the general case requires access to all past values of the solution in the time span required by the delays. To implement this, we begin by specifying a minimum delay (called min_delay), which is the step size of the simulation. The simulation step size is called min_delay because the minimum value among the connection delays is an upper bound on the step size.

For example, in the system:

$$\begin{array}{l} \frac{dx\left(t\right)}{dt}=y\left(t-0.2\right),\;\frac{dy\left(t\right)}{dt}=-x\left(t-0.5\right); \end{array}$$

there is a minimum delay of 0.2 s, and to simulate the system we need to store values of *x* spanning the last 0.5 s, and values of *y* spanning the last 0.2 s. Using this we can advance the state from time *t* to time *t* + 0.2 using a standard ODE solver. The simulation can thus proceed with time steps of size 0.2, although the numerical solver may calculate many states at intermediate time points for each time step. In order to simplify the numerics a further requirement is that all delays are multiples of min_delay, so for this example min_delay could actually be 0.1, or some other common divisor of 0.2 and 0.5. Furthermore, the user may set a smaller value of min_delay in order to enhance numerical precision (see Appendix B).

In Draculab every unit has a buffer with past activation values spanning a time interval equal to the longest delay in the unit's projections. The network parameter min_buff_size in the first example of section 2 indicates how many values are stored for a min_delay time period in all buffers; the number of past activation values in a unit's buffer depends on how many min_delay time periods it spans. If unit *A* sends a projection to unit *B*, when *B* is updating its state it will request unit *A* for any past values that it requires. Unit *A* will respond to these requests by using linear interpolation on the values stored in its buffer. It is possible to specify other interpolation methods (see section 9 and Appendix B). Moreover, for some implementations of ODE integration (such as the forward Euler example above) interpolation is not required (see section 5).

**Figure 5:**
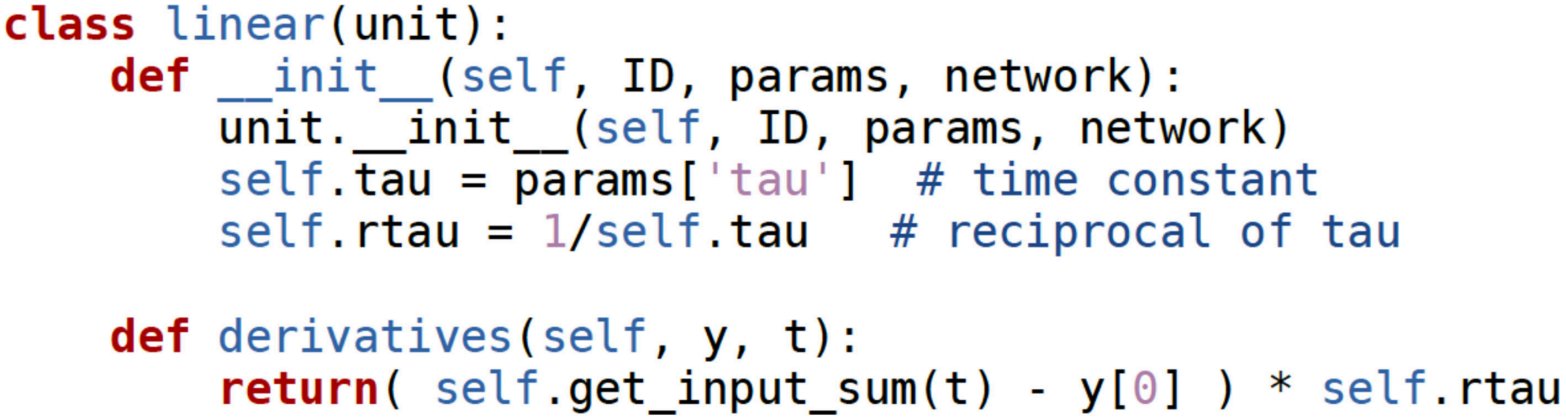
The **linear** unit class. Docstring comments were removed for brevity.

In Draculab this is transparent to a user writing a new unit model. All the user needs to do is to create a new class for their custom unit, and to have that new class inherit from the unit class. This will provide the new class with the methods that handle buffers and integration, which use Cython optimizations and Scipy's odeint numerical solver by default; a different solver can be specified for a unit with the integ_meth attribute of its parameter dictionary. The new unit class only needs to specify a constructor (__init__) to initialize its variables, and a derivatives(y, t) method, that will tell the numerical ODE solver what's the derivative of the unit's firing rate at time *t*, given that the current state is *y*. Figure 5 provides the code implementing a simple linear unit, where this can be seen explicitly. All the functionality of the unit is implemented using 3 lines.

**Figure 6:**
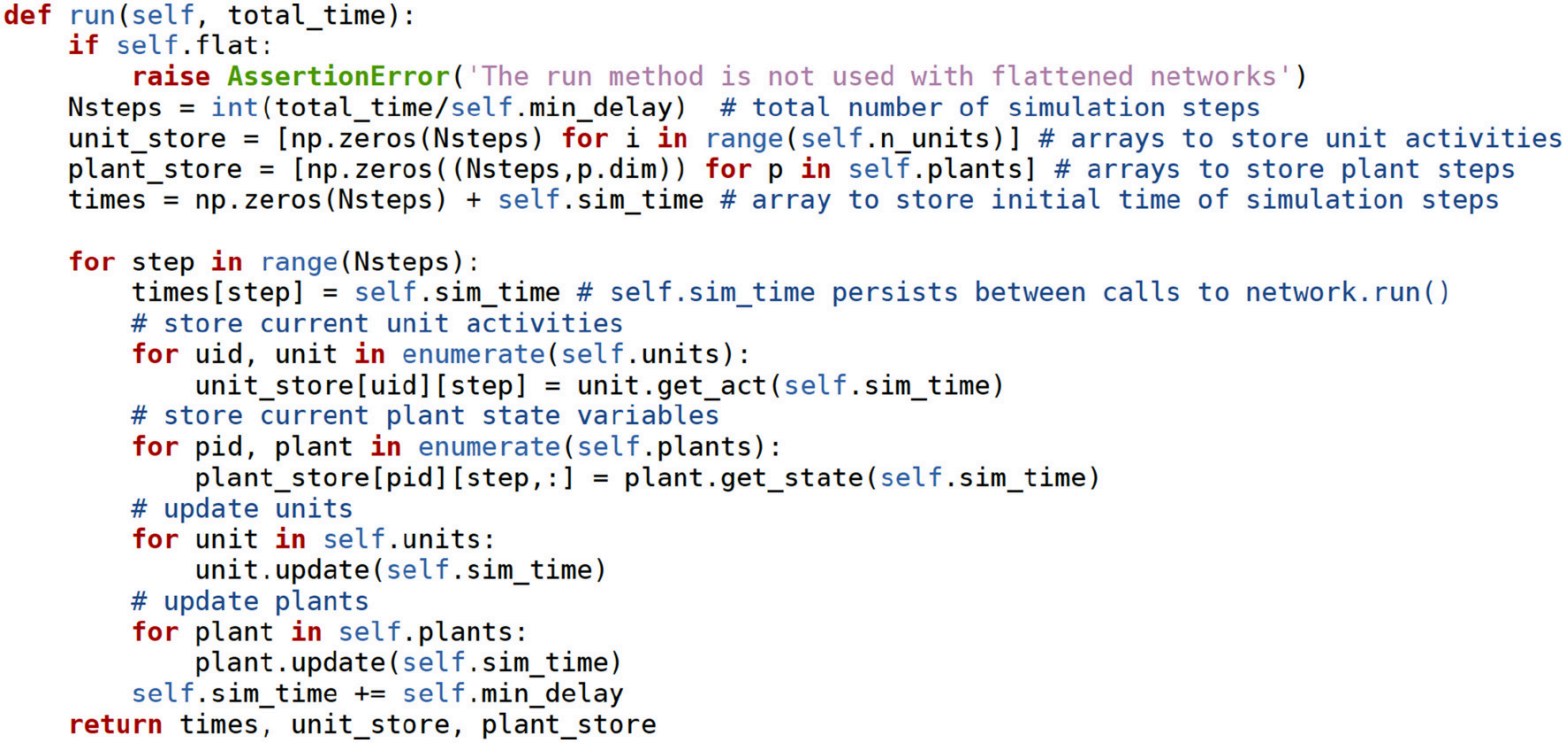
The **network.run** method receives the time to simulate as an argument, and advances the simulation taking steps of **min_delay** length. At each step the units, synapses, and plants use their own methods to advance their state variables. The values of the state variables and of the source units are stored in the **unit_store** and **plant_store** Numpy arrays. **np** stands for the **numpy** module. Docstring comments removed for brevity.

At this point it is also possible to read the method used to run the simulations (network.run), whose source code is in Figure 6. The reader is encouraged to have a look at this short example.

A unit has a single output, but it may many more dynamical variables used to assist the computation of synaptic dynamics and its own firing rate. The firing rate of the unit is a *fast* variable, integrated with the odeint solver. Units may have other variables, often evolving on slower timescales, updated every min_delay time units with a user defined *update* method. These variables are usually required to compute synaptic plasticity. For example, in the Law and Cooper version of the BCM plasticity rule (Law and Cooper, 1994) the synaptic weights evolve according to the equation:

$$\begin{array}{l} w^{\prime }=\alpha x_{pre}x_{post}\left(x_{post}-\theta \right)/\theta \end{array}$$

where *x*_*pre*_ and *x*_*post*_ are the presynaptic and postsynaptic firing rates, α is the learning rate, and θ is the average of the squared postsynaptic activity. To avoid duplicate calculations, a variable like θ should be computed by the units rather than by the synapse objects. In this case, θ can be obtained using a first order low-pass filter on *x*_*post*_, and when this is implemented the unit updates two state variables: its firing rate, and the low-pass filtered value, which is a synaptic *requirement*.

“Requirements” are updated every time the unit.update function is called. This is also the function that updates the firing rate and the unit's buffer. unit.update handles requirements by calling the function pre_syn_update, which invokes all functions required for this end. If the user wants to write a plasticity rule that uses a value such as the sum of inputs, or something more exotic, this can be done by writing a function that updates the required value, and by including that function among the ones called by pre_syn_update.

A design convention in Draculab is that synapses do not have buffers to store past activation values, as units do. Synapses are updated once per simulation step, and as far as the unit's firing rate dynamics are concerned, the synaptic weights are constant during this min_delay period. This comes from the observation that the changes in synaptic weights tend to be much slower than the changes in firing rate, so we might as well update them using a simple integration rule (such as forward Euler) explicitly written in the synapse's update method. This can bring significant gains in efficiency. For example, the bcm_synapse class implements the BCM learning rule described above, and has the following update method, getting called once per simulation step by the unit.update method of its postsynaptic unit.


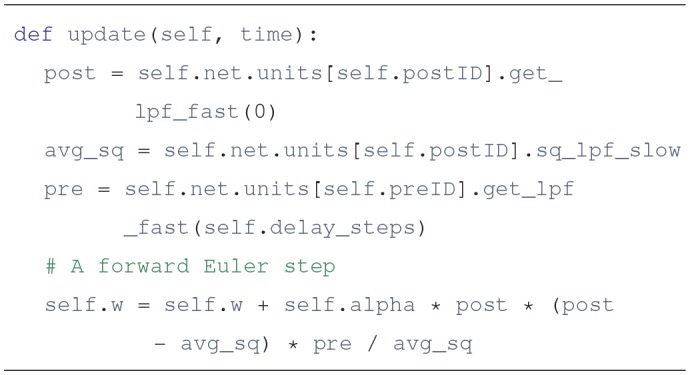


There are some clarifications to make about this piece of code:

- self refers to the synapse object, self.net to the network object, and sel.net.units is a list that contains all the units in the network. The index of a unit in this list is its unique identifier (ID). The synapse keeps the identifiers of its postsynaptic unit (postID), and its presynaptic unit (preID). The value alpha is the length of the simulation step times a learning rate.
- This implementation of the learning rule does not really use the pre- and postsynaptic firing rates. Instead it applies low-pass filters with a fast time constant to the firing rates, and uses that in the equations. A version of the firing rate passed through a first order low-pass filter is a standard synaptic requirement in Draculab, as it can promote stability in continuous-time systems. Draculab uses the analytical solution of the filter's differential equation in order to implement an explicit solver. A unit's lpf_fast value is its “fast” low-pass filtered activity, and a unit's get_lpf_fast(n) method returns the lpf_fast value as it was n simulation steps before (to account for transmission delays).

Plants are very similar to units, but they may have more than one state variable that gets updated by the odeint solver. Units can send projections to plants, and plants can send projections to units, in both case mediated by synapses. Direct connections between plants is not supported.

As with units, to create a new plant a user has to define a class. This class only requires __init__ and derivatives methods to be defined.

From the information so far it is possible to get a general outline of how a Draculab simulation proceeds when network.run (Figure 6) is executed:

1. The network object stores all unit and plant values from the previous step.
2. The network object requests all units and plants update their state.
3. In unit.update:
  - Units first update the content of their buffers from the current simulation time t, to t+min_delay, using the values in the buffers of all other units, and their own integration method (odeint by default).
  - Units run pre_syn_update, which updates the values of all their requirements.
  - Units call the update method of all synapses providing them inputs. This updates their weights.
4. In plant.update the plants update the content of their buffers.

The next section brings a little more detail to this process.

## 4. The Essential Draculab Data Structures

Draculab uses four basic classes to run simulations: network, unit, synapse, and plant. These comprise the core simulator. Each instance of the network class is a full neural network with its own set of units, synapses, and plants, which the network stores in 3 lists named units, syns, and plants. As mentioned in section 3, each unit has an identifier ID, which is its index in the units list.

Representing the connectivity information in a network with no delays can be done with a weight matrix. Furthermore, the product of the weight matrix times the vector of unit activities conveniently provides the sum of inputs times their synaptic weights for all units. This is not so straightforward when there are connection delays, so a new approach is needed. Draculab provides two different options to represent connections. The first one, to be described in the next section, uses advanced Numpy array indexing so that each unit has an index representing all its inputs and their delays. The second one, to be described in this section, solves all connectivity issues using 3 lists: the aforementioned syns, and two other lists called delays and act.

Each element in syns is another list. The list syns[i] contains all the synapses from the projections received by the unit with ID=i. syns[i][j] is the synapse object for the j-th connection to the i-th unit. The delays list has the same structure: delays[i][j] is the delay of the j-th connection to the i-th unit. act has also this structure, but it requires some clarifications.

Every unit has a get_act(time) method that provides its activity (firing rate) as a function of time. This method obtains the activity using interpolation on the values of the unit's buffer. The value act[i][j] contains a reference to the get_act method of the presynaptic unit for the j-th connection to unit i.

Using these three lists it is simple to get the j-th input to unit i at time t: it is act[i][j](t - delays[i][j]). To further illustrate this, here is a plausible implementation of the unit.get_input_sum method, which provides the sum of inputs times their synaptic weights:


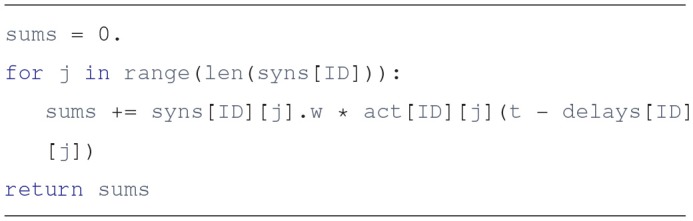


The actual implementation uses a list comprehension, and may not be as readable to some, but it gets everything done in a single line:


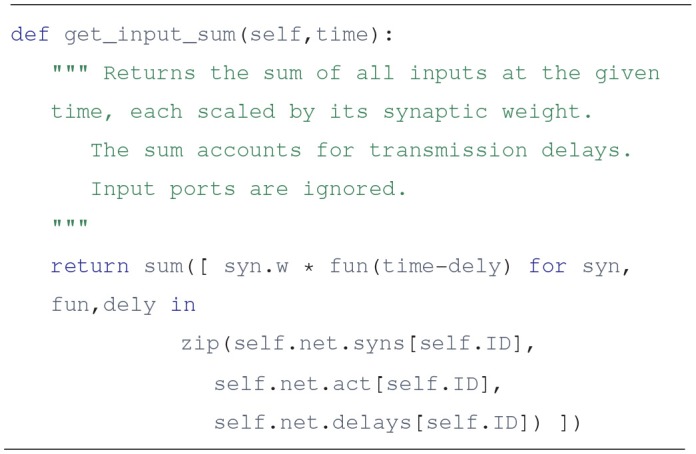


Lists can offer a clear solution to the problem of connectivity. It is not the fastest way, but it agrees with the principles of Python and Draculab. Simple is better than complex. Still, sometimes we may want to trade some simplicity for the sake of speed, so the approach of section 5 is offered.

## 5. In Search for Speed

The architecture described so far was created with simplicity and flexibility in mind, but it would be great if it could be fast too. Speed, however, is limited because the get_input_sum method shown above relies on Python data structures. In particular, the connectivity structure is described by the delays and act nested lists. The speed bottleneck imposed by this can be broken through a design we call the *flat network*.

In a flat network the data of all the unit buffers is placed in a single 2-dimensional numpy array in the network class, called acts. It could be said that all the unit buffers are *flattened* into acts. The unit class still retains its buffer attribute, but it now becomes a *view* of a slice of the acts array. Being a view means that the buffer of a unit is a numpy array that uses the same memory addresses as its corresponding entries in the acts array.

With the past activation data for all units contained in the acts array, connectivity and delays can be represented by having a structured index in each unit, so that when applied to acts, it retrieves all the input values that the unit needs to calculate its input sum at several time points. In other words, if a unit has an idx index, then acts[idx] will return an array with all the inputs the unit received during a min_delay time period. In the current implementation, if the unit has *m* inputs, then acts[idx] has *m* rows and min_buff_size columns. Using this array is straightforward to calculate the input sums to the unit.

To the user all of this is transparent. Creating a flat network is identical to creating a normal Draculab network. The difference is that the run method used to start simulations is substituted by the flat_run method, which automatically uses the flat network. The first thing that flat_run does is to call the network.flatten method, which moves the unit buffers into the acts array, along with other preparations. The rest of flat_run is very similar to run except for the method that updates the units' activity.

To a developer writing a Draculab unit model, there is a small difference between writing it for a regular or a flat network. As mentioned before, for the regular network the user needs to write init and derivatives methods. For a flat network a dt_fun method must be written instead of derivatives. The difference between these two is that whereas derivatives retrieves the input sum using the get_input_sum method, dt_fun retrieves the input sum directly from an array in the unit called inp_sum. For example, here is the dt_fun method for linear units:


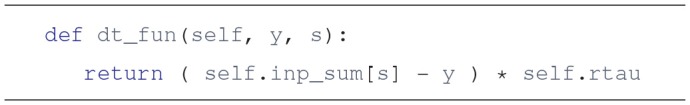


As this suggests, for most developers using a flat network will not bring an increase in complexity. Flat networks are more challenging only for those wanting to write new integration methods for the unit, as this entails advanced array indexing. Currently, all integration methods used for flat networks use a fixed step size, because with these it is not necessary to interpolate in order to obtain the required past activation values. There are versions of the “Euler,” “Euler-Maruyama,” and “Exponential Euler” integration methods using this scheme. It should be noted, however, that flat networks can work with solvers that use variable step sizes. In fact, the default solver for plants in flat networks is Scipy's solve_ivp.

The reduction in execution time from using a flat, non-interpolating integration method is dependent on the specific structure of the network, but it is common to observe that a flat network is at least 3 times faster than the analogous regular network.

Readers who have progressed this far have already peeked at the heart of Draculab, and should have no major trouble in creating custom models and tweaking things after going through the tutorials.

## 6. Noise

Noise can be an important component of neural computations (Destexhe and Contreras, 2006; Swain and Longtin, 2006), and is sometimes part of rate models (Hahne et al., 2017).

External noise present in the inputs to a unit is straightforward to implement using source units. A source unit that provides noise input can be created by assigning it a function that produces random values from a particular distribution. It is recommended to use the numpy.random library for this purpose, because Draculab uses this library for its own random routines (e.g., to create random initial weights or random connectivity). Thus, if numpy.random is used to provide noisy inputs then a single seed initialization [e.g., an instruction like numpy.random.seed(546789054)] permits to reproduce the simulation with the same random values.

Intrinsic noise can also be added to the unit models, turning their ODEs into Langevin equations. Specifically, if the equation of the model is:

$$\begin{array}{l} x^{\prime }\left(t\right)=f\left(x,t\right) \end{array}$$

it can be turned into the stochastic differential equation:

$$\begin{array}{l} dx\left(t\right)=f\left(x,t\right)dt+\sigma dW\left(t\right) \end{array}$$

where *W*(*t*) denotes a Wiener process with unit variance.

Turning the ODE into an SDE can be done by substituting the odeint numerical solver by a stochastic integrator. This can be done by specifying a different integration method with the integ_meth entry of the unit's parameter dictionary. Currently two stochastic integrators (Euler-Maruyama and “stochastic exponential Euler”) are available, as Cython utilities for regular networks, and as methods of the unit class for flat networks.

It is easy to create simulations where different units have different integration methods. The one restriction is that all integration methods must be either for regular, or for flat networks. The integration methods have different implementations for these two cases, and the network can't mix flat and regular methods.

## 7. Beyond the Core Simulator

Writing down all the parameters and connectivity details of a complex network can be a daunting task, even with a simulator handling the basic creation and simulation routines. Draculab's general approach is to separate the core simulator from the tools used to make simulations easier to write and visualize.

Python provides enough power so that individual users can create configuration and visualization solutions according to the model they are writing. Nevertheless, we offer a few tools to make this easier. First and foremost is the topology module, which allows to create spatially structured connections. The second are the ei_net and ei_network modules. These are a collection of Python classes and methods to quickly create a particular type of networks with standard parameters, and to visualize the simulation results using Matplotlib.

Draculab's topology model is inspired in NEST's topology module^1^. Although the tools to define connection profiles are similar, there are some key differences, the main one being the Draculab's topology module does not use layer objects.

The function used to create structured connections is called topo_connect. As with network.connect, the first argument to topo_connect is a list with the IDs of the units sending the connections, and the second argument is a list with the IDs of the units receiving the connections. Unlike network.connect, all units must have a coordinates attribute that describes their spatial location. The third argument is a connection specification dictionary, which determines the probability of connection (and optionally strength of synaptic weights) between any two units based on their coordinates. The fourth argument is a synapse specification dictionary, with the same format as in network.connect.

Since topo_connect expects units with spatial coordinates, the topology module also includes a method to create them, called create_group. This method resembles network.create, but it receives an extra geometry parameters dictionary as a way to specify how many units to create, and where to put them. Currently create_group can only create flat two-dimensional layers with units in a grid or in random arrangements. Still, both topo_connect and create_group are written to support a possible expansion to 3D coordinates. An alternative to create_group is to provide a list with coordinates to network.create.

The ei_net and ei_network modules have classes that can build networks with 3 populations: one with excitatory units, one with inhibitory units, and one with source units. All parameter dictionaries are written into these classes, so networks can be created with a single instruction. After creation the default parameters can be modified (these modifications get automatically logged), and then the network can be *built*. At this point simulations can be run and results can be visualized, either with plots or with animations. The example network from section 2.2 used this approach at a smaller scale. Specifying simulations by modifying a standard set of parameters greatly reduces their description length, and the possibility of errors.

The difference between ei_net and ei_network is that ei_net specifies a single “layer” object, whereas ei_network has tools to create and connect several “layer” elements. Both modules provide solutions to the most common tasks in neural network simulations, including parameter specification, input configuration, storage, documentation, visualization of connections, and visualization of results. These two modules implement a particular type of network for a particular type of simulation, but it is expected that individual users can adapt these tools to their own needs. This complements various other choices that users have, such as NeuroTools and Sumatra (http://neuralensemble.org/) for simulation management and analysis, and scikit-learn (https://scikit-learn.org/stable/) for machine learning analytics.

It is easy to program a plant to interact with the network, as long as the plant is simple, such as a planar arm. Things are harder when the plant is more complex, such as the model of a particular robot, and things are downright hard if the actuator has to interact with a virtual environment. Fortunately there are physics simulators written with these considerations in mind (Ivaldi et al., 2014). When using an external physics simulator the plant object can stop being an implementation of the physics simulation, and become an interface to the physics simulator. Writing a simulation where Draculab interacts with a physics simulator may require considerable expertise.

A relatively simple option to integrate a Draculab controller in a virtual environment is provided by the HBP neurorobotics platform (NRP) (Hinkel et al., 2017) (https://neurorobotics.net). The NRP calls a Python *transfer function* on each simulation step, and Draculab can be used in it. In part this is possible because Draculab's core is compatible with Python 2.7, although it was developed with Python 3.5.

The obvious drawback of using the NRP is that the user needs to understand how to program it. Also, a basic understanding of the services provided by the Robot Operating System (https://www.ros.org/) may be necessary in some cases. Physics simulators make it easier to produce a virtual environment, but they bring a new learning curve.

## 8. Alternatives to Draculab

There are excellent simulators for firing rate networks (Aisa et al., 2008; Cofer et al., 2010; Rougier and Fix, 2012; Bekolay et al., 2014; Vitay et al., 2015; Tosi and Yoshimi, 2016), but it is unusual to find rate simulators that take into account connection delays. To the authors' knowledge there are currently three simulators with this capability: The Virtual Brain (Sanz Leon et al., 2013) (https://www.thevirtualbrain.org), MIIND (de Kamps et al., 2008) (http://miind.sourceforge.net/index.html), and NEST (Diesmann and Gewaltig, 2001) (http://www.nest-simulator.org/).

The Virtual Brain is a simulation platform with a graphical user interface. Among other things, this platform includes data management and analysis functionality so that models with realistic large-scale connectivity of identified brain regions can be compared with brain scanning data such as fMRI, MEG, or EEG. Although it is an open source project, it does not appear feasible for normal users to add their own custom models or to integrate the platform with biomechanical simulations.

MIIND is a very flexible framework for simulation of neural networks, consisting of a C++ library that can be used to describe network components in a modular fashion, and to perform simulations. Perhaps the most distinctive feature of MIIND is the support it offers for population density techniques, which use differential equations to model the distribution of states for a neural population. This can describe the behavior of large populations of leaky-integrate-and-fire neurons at a fraction of the computational cost.

The use of connection delays is not explicitly mentioned in the MIIND 2008 paper (de Kamps et al., 2008), or in the current version of the tutorial^2^, but a procedure to introduce them is present in the API documentation, and simulations with connection delays are already producing results (personal communication).

NEST is a simulator for spiking neural networks, but it has recently incorporated firing rate models (Hahne et al., 2017) for the benefit of easy validation of mean-field approaches, and at some point in the future to allow multi-scale modeling with both spiking and rate neurons. Over the years NEST has become a mature software project, with a large C++ codebase capable of handling massive parallel simulations on supercomputers. Learning how to create new neuron or synapse models is thus a significant endeavor, but the resulting implementations can take advantage of NEST's infrastructure.

Considering that NEST is often taken as a reference for the performance of spiking neural models, it seems appropriate to use its rate models to assess Draculab's efficiency. NEST's rate models still lack features such as sources of temporally varying inputs or plastic synapses, but we expect that development will continue to move forward. At the time of this writing continuous-time inputs to rate models were soon to be released (personal communication).

As an initial exploration the rate_neuron_dm example distributed with the NEST version 2.16 codebase was modified by changing its instantaneous connections to delayed connections with a 4 millisecond delay. The elapsed simulation time was compared with that of an equivalent Draculab implementation (Appendix A). Both simulations showed the same response in the absence of noise. When the NEST file is modified only by placing a delay in the connections the execution time rises sharply, as seen in Table 1. Such a result is restricted to networks with small *resolution* values. This resolution is a kernel parameter in NEST, specifying how much precision is available in the output. The closest parameter in Draculab may be the minimum delay divided by the minimum buffer size, which is matched with NEST's resolution for the comparisons of this section. Reducing the resolution in the rate_neuron_dm example dramatically brought down the execution time for NEST (Table 1).

**Table 1:** Simulation times for the rate_neuron_dm model.

resolution	0.001	0.01
Draculab time	5.8 s	1.0 s
NEST time	92.5 s	1.5 s

**Figure 7:**
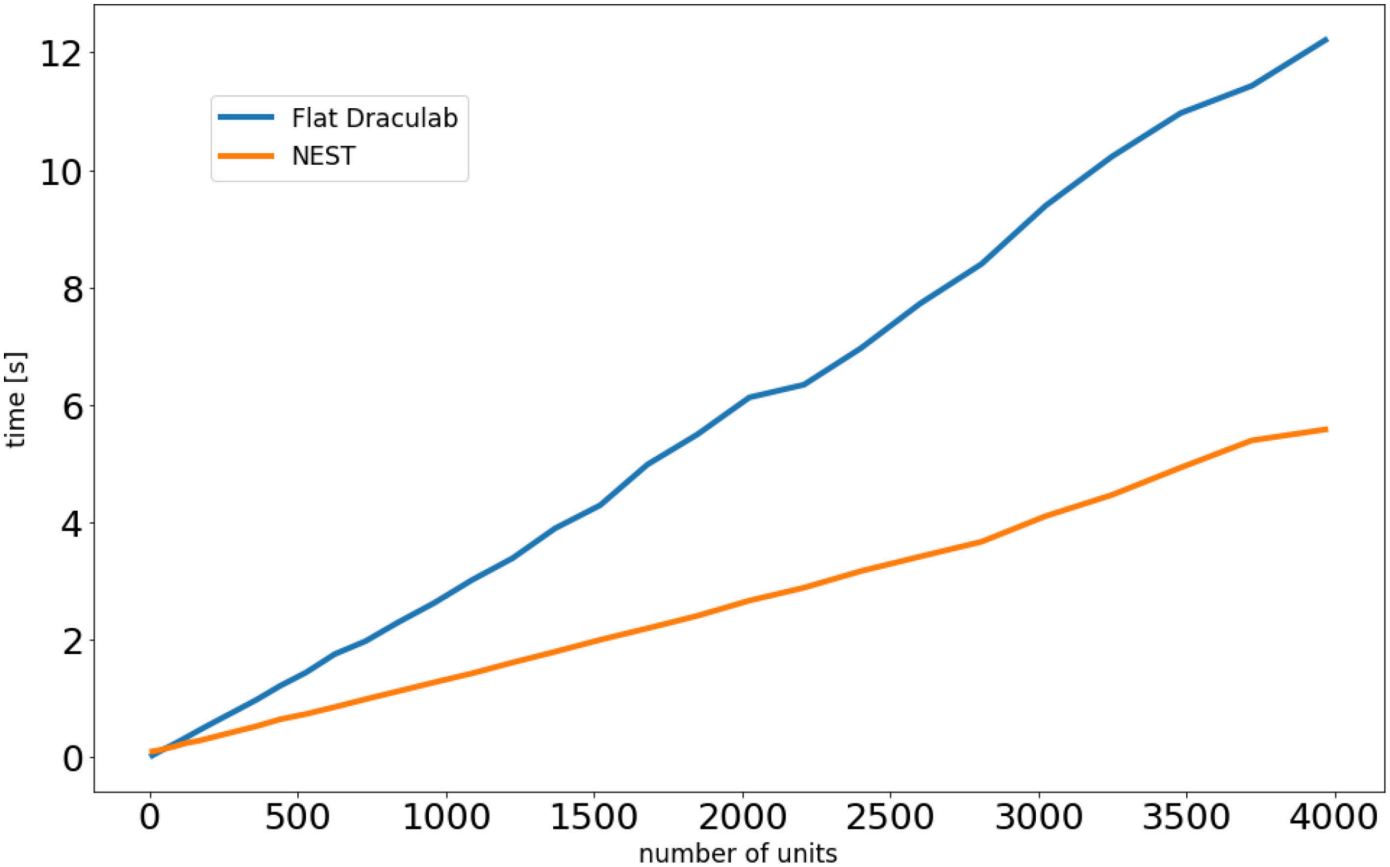
Simulation times as a function of the number of units.

Draculab is not designed for large networks, whereas NEST is highly adapted for this. To explore how computation times scale with network size both simulators were programmed with equivalent network simulations (Appendix A). For this comparison, Draculab used a flattened network. Figure 7 shows the results. For small networks both simulators perform similarly, but NEST clearly scales better for large networks.

It should be noted that NEST, MIIND, and Draculab offer complementary approaches. Draculab's approachable architecture and simple Python interface permits researchers to quickly create and test prototypes, going through several iterations until the right properties are found. When the need for large or fast simulations arises, one of the C++ alternatives can be used. NEST is a great choice for users who want to run spiking and rate simulations side by side. On the other hand MIIND may be the best option when population density techniques are appropriate. The Virtual Brain is an easy choice when comparison with brain scanning data is required.

## 9. Discussion

The inclusion of connection delays in a firing rate simulator merits some consideration.

Research in firing rate neural networks has brought a large number of results. Many of the early results produced the Parallel Distributed Processing Paradigm (Rumelhart and Mcclelland, 1986) (including backpropagation and Boltzmann machines), eventually leading to the more recent “deep learning” trend (Schmidhuber, 2015). Because of their feedforward connectivity and lack of continuous dynamics these models have deviated from biological plausibility, and it is unclear whether their computation style is anything like biological brains. There is also a wealth of firing rate models that represent cognitive processes (e.g., Carpenter and Grossberg, 1991; O'Reilly, 1998; Mastebroek et al., 2001; Eliasmith, 2013). Although these latter models aim for biological plausibility, virtually all of them lack connection delays.

The omission of connection delays is not surprising. Mathematically it moves the models from the realm of linear algebra into functional analysis, with the consequent increase in complexity. Computationally it forces the storage of past activation values for all units, with the consequent increase in computational costs. And then again, delays may not change the network's dynamics, considering that in the brain delays can be as short as one millisecond. Indeed oftentimes delays in the millisecond range do not change the network's dynamics, but it can't be denied that this is not always the case.

In the realm of spiking networks many arguments have been presented to support the importance of precise timing (Rieke, 1999), in which connection delays play a crucial part (e.g., Izhikevich, 2006). On the other hand, the hypothesis of firing rate coding seems to be the opposite of precise timing playing an important role. This last characterization of firing rate is incorrect. When a network's dynamical system has non-linearities such as the ones creating a separatrix in the phase plane (Ermentrout and Terman, 2010), minute changes in the firing rate can create very different dynamical trajectories, and this translates in minute changes in timing causing very different results. This compounds when the firing rate can vary quickly, (e.g., when it comes from a population average, or when its time window for averaging is short). Precise timing can be important in firing rate networks, it's just that the rate is invariant to permutations in the identity of the neuron producing each individual spike (in population averages).

It is thus clear that in principle the connection delays can play an important role in firing rate networks too, and this role comes into focus when simulations aim to produce biologically plausible closed-loop control in continuous time. Firstly, it is common knowledge in control theory that the presence of delays in closed-loop feedback control imposes fundamental limitations on performance (Mirkin and Palmor, 2005), and can create chaos even on first order systems (Mackey and Glass, 1977). These performance limitations have prompted the rise of predictive control. As an example of how this is important, the cerebellum has been hypothesized to be a predictor that permits motor control signals with millisecond precision despite delays in the sensorimotor loop (Wolpert et al., 1998; Dean and Porrill, 2008; Imamizu and Kawato, 2009; Verduzco-Flores and O'Reilly, 2015). Secondly, firing rate coding is found in sensory receptors (Ahissar et al., 2000; Salinas et al., 2000; Butts and Goldman, 2006), and is the way that motor neurons control contractions of skeletal muscle (Botelho, 1955; Ali et al., 1970; MilnerBrown et al., 1973). Rate coding is thus often found in the interaction of the vertebrate nervous system with its sensors and with its actuators.

This type of considerations are not new to neural modelers. For example, there were plans to provide built-in time-delayed connections in the DANA simulator in order to provide biological realism (Rougier and Fix, 2012), and the NEST simulator is developing its own infrastructure to validate rate models (Hahne et al., 2017). Draculab is also a response to this need, but it also has an unusual aim: that most users can work at the source code level when they need something completely different. The extent to which this can be done depends on the clarity of the design, as well as the user's sophistication level. It is plausible that Python is clear enough, Draculab is simple enough, and researchers in computational neuroscience are capable enough.

Draculab embodies particular research principles. That neural networks are more interesting when they can implement cognitive functions, and when they can do it with biological plausibility. That cognitive models are more illuminating when they span the action-perception loop. Those principles are the source of Draculab's characteristics, such as rate models, delays, and plants.

Future development plans include:

- performance optimizations (without adding too much complexity to the simulator core),
- improvements to the unit tests,
- improvements to the documentation,
- improving the interface with the neurorobotics platform.

Regarding this last point, the HBP neurorobotics platform will provide a new API to integrate it with neural simulators (personal communication). This may be better than the current method, which embeds Draculab in a transfer function. Developers of the neurorobotics platform have also begun translating several OpenSim (Delp et al., 2007) (http://opensim.stanford.edu/) models into the NRP, making it appropriate for biomechanic simulations.

A final point to make is in regard to the precision and accuracy of the solutions. Precision is of course dependent on the solver and the parameters used. The simulation step size given by min_delay is always important. In the case of the odeint and solve_ivp solvers the rtol and atol parameters become relevant. When using the forward Euler method, the step size is min_delay/min_buff_size. Moreover, the interpolation method used to retrieve past values can also affect precision. Draculab can use Scipy's interp1d function for this end by modifying two lines in the units.py file, but this usually comes with a reduction in speed.

It is not always obvious how the precision of a simulation will be altered depending on parameter settings. To help users investigate this, an IPython notebook was created (the file tests/compare_accuracy.ipynb of the distribution), where it is easy to test various simulator configurations. As an initial guide, the results of running a network with high-precision parameters were compared against the results when performing various parameter modifications. This is reported in Appendix B.

There is currently no agreed standard to test the accuracy of neural simulators. In the absence of a common benchmark, the approach taken to verify that solutions were correct was to compare runs of the same network in different simulators. In particular, the same network was simulated using XPP (Ermentrout, 2012) and Simulink (https://www.mathworks.com/products/simulink.html), and the resulting unit activities were compared with Draculab. Results were almost identical for the three simulators. This can be found among Draculab's unit tests. Additionally, the performance comparison with NEST (section 8) provided an opportunity to compare Draculab's and NEST's output. Results generally agree, although there are small differences for networks with many units, perhaps from differences in the way that initial conditions are specified.

Draculab's source code can be obtained from this repository: https://gitlab.com/sergio.verduzco/draculab It is distributed freely under the GNU GPLv3 license, with the hope that it can become a valuable tool for researchers who find that firing rate models with delays and closed-loop control hit a sweet spot of biological plausibility, technical feasibility, and scientific insight.

## Data Availability

The source code created for this study can be found in the Draculab repository:https://gitlab.com/sergio.verduzco/draculab

## Author Contributions

SV-F created the software, and wrote the first draft of the manuscript. ED supervised the work, and performed manuscript revisions.

### Conflict of Interest Statement

The authors declare that the research was conducted in the absence of any commercial or financial relationships that could be construed as a potential conflict of interest.

## Footnotes

^1^http://www.nest-simulator.org/introduction-to-pynest/part-4-topologically-structured-networks/

^2^http://miind.sourceforge.net/tutorial.pdf
