# Supplementary material for: Draculab: A Python Simulator for Firing Rate Neural Networks With Delayed Adaptive Connections
Source: Front Neuroinform. 2019 Apr 2;13:18. doi: 10.3389/fninf.2019.00018 (PMC6454197; doi:10.3389/fninf.2019.00018)
Supplement: Supplementary file 1 [file Data_Sheet_1.pdf]

Draculab: A Python simulator for firing rate neural networks with delayed adaptive connections. Appendix.

Sergio Verduzco-Flores, Erik De Schutter

1 Appendix A

Comparing with NEST

The file `rate_neuron_dm.py` distributed with the source code of NEST 2.16 was modified to use connection delays, and to store the data for the simulations with no noise. The resulting code can be found in the `tests/rate_neuron_dm_mod.py` file in the repository. A Draculab unit model that replicates the dynamics of the `lin_rate_ipn` model was created, and specified to use the stochastic exponential Euler numerical solver when its parameter `lambda` was positive. The code from `rate_neuron_dm.py` was modified to run simulations with this model instead. The resulting code is in the `tests/rate_neuron_dm_dracu.py` file.

The comparison leading to figure 7 of the main text was produced with a Jupyter notebook found in `tests/rate_neuron_dm_comp.ipynb`. In brief, the networks compared consisted of squared grids of units whose row and column size were gradually increased. Each unit was connected to its 8 neighbours using delayed connections. A sinusoidal input was applied to half of the units. Draculab was loaded using Cython, which can be done using two commands in the Jupyter notebook (details are in the tutorial).

The simulation was run on a personal workstation with an Intel Core i9-7900X processor. Simulations used a single thread.

2 Appendix B

Effect of parameter modifications on precision and simulation times

Using the Jupyter notebook in `tests/compare_accuracy.ipynb` a sequence of simulations were run. First, a high-precision simulation was performed with:

- Cubic spline interpolation for the buffer values.
- A relatively large minimum buffer size = 40.

- Use of the `odeint` solver with `rtol = 1e-5, atol = 1e-5`.
- A small `min_delay` (minimum delay) value (0.001).

Each one of these factors were varied. For each variation, the activities of all units for all points in time were substracted from the corresponding activities in the high-precision results, and the maximum among the absolute values of all these differences is reported as the maximum difference *max diff.* The mean of the set of absolute difference values is reported as *mean diff.* The simulation time for each variation is reported as T.

The first variation (*V1a*) changes the configuration of `interp1d` to use linear interpolation, instead of cubic splines. The next variation (*V1b*) uses Draculab's own linear interpolator. Results between variations V1a and V1b may differ because the `odeint` integrator often requests values outside of the buffer's ranage. `interp1d` was configured to handle this with extrapolation, whereas the Draculab solver provides a different value.

Variations V2a and V2b change the value of the minimum buffer size to 20 and 5, respectively.

Variations 3a and 3b change the `rtol` and `atol` values to .001, and 0.1 respectively. Variation 3c replaces the `odeint` integrator for the `euler` method. Variation 3d uses the `euler` integrator and Draculab's linear interpolation. Variation 3e uses a flat network with `euler` integration.

Variation 4a increases `min_delay` to 0.02, and 4b increases it to 0.2 .

Variation 5a is meant to show how not much precision is lost when using a flat network with Euler integration that keeps the parameters of the high-precision configuration, and on the other hand simulation times are greatly reduced. Variation 5b shows how, on the other hand, Euler integration can become unstable if the step size `min_delay/min_buff_size` is not controlled.

The result of simulating all variations is summarized in table 1. Results are not reported for V5b, since the simulation was unstable.

variation	max diff	mean diff	T
V1a	0.000091	0.000001	178.89
V1b	0.0154	0.000068	14.997
V2a	0.000104	0.000001	177.83
V2b	0.000483	0.000008	180.77
V3a	0.003741	0.000034	168.96
V3b	0.022	0.000089	165.6
V3c	0.001637	0.000012	835.56
V3d	0.014383	0.000051	55.04
V3e	0.004	0.000019	10.12
V4a	0.519	0.004	76.0
V4b	0.982	0.0767	22.13
V5a	0.00159	0.000005	52.25

Table 1: Result of running variations on a high-precision configuration.
